# Management of Refractory Facial Melasma Using the NewMelan Topical Protocol Following Multiple Unsuccessful Treatment Modalities: A Case Report

**DOI:** 10.7759/cureus.117880

**Published:** 2026-10-13

**Authors:** Sikiu Krulig

**Affiliations:** 1 Dentistry, Oralmed, Miami, USA

**Keywords:** depigmentation therapy, facial hyperpigmentation, melasma, newmelan, orofacial aesthetics, photoprotection, refractory melasma, skin pigmentation, sun exposure, topical treatment

## Abstract

Melasma is a chronic and recurrent facial hyperpigmentation disorder that can be challenging to manage, particularly in individuals with frequent sun exposure and a history of unsuccessful treatment. This case describes a 37-year-old medically healthy woman with persistent facial melasma and substantial occupational sun exposure related to her work in a beach environment. The condition had become a significant esthetic concern and negatively affected her self-confidence. The patient reported multiple previous treatment attempts, including chemical peels, laser-based procedures, and topical depigmenting therapy with hydroquinone, tretinoin, and niacinamide, without satisfactory improvement. Following clinical evaluation and the exclusion of a known hormonal or relevant systemic disorder based on the patient’s medical history, an individualized topical protocol using NewMelan PRO (Krulig International Inc., Pembroke Pines, Florida, United States) and NewMelan Day & Night (Krulig International Inc., Pembroke Pines, Florida, United States) was initiated without concurrent laser therapy, chemical peeling, microneedling, injectable treatment, or additional depigmenting agents. NewMelan PRO was initially applied as a thin, full-face mask, followed by a structured regimen of NewMelan Day & Night with progressively adjusted application frequency and subsequent maintenance therapy. Progressive improvement in facial hyperpigmentation was observed during treatment. At approximately 10 days, qualitative clinical examination and comparison with the baseline photographs indicated a visible reduction in the intensity and extent of pigmentation, with further visible improvement documented at the 15 day clinical assessment. The patient continued topical NewMelan (Krulig International Inc., Pembroke Pines, Florida, United States) therapy thereafter as part of the treatment and maintenance regimen. No validated standardized scoring system, colorimetry, or spectrophotometry was used. The patient reported high satisfaction with the clinical outcome. This case documents short-term clinical improvement in persistent facial melasma during a structured NewMelan topical protocol following several previously unsuccessful treatment modalities. However, as a single patient observation involving nonstandardized clinical photography and short-term assessment, the findings do not establish causality, comparative efficacy, or long-term treatment durability. This case highlights the importance of individualized management, careful assessment of previous therapies, continued maintenance treatment, and photoprotection in patients with persistent melasma.

## Introduction

Melasma is a chronic, acquired disorder of hyperpigmentation that typically presents as symmetrical brown to gray-brown macules and patches involving sun-exposed areas of the face. It occurs predominantly in women and has a multifactorial pathogenesis involving genetic susceptibility, ultraviolet and visible light exposure, hormonal influences, and alterations in melanogenesis and the cutaneous microenvironment [1]. Although melasma is benign, its visible facial distribution, chronicity, and recurrent course can make long-term management challenging.

Management of melasma frequently requires multiple therapeutic approaches. Oral tranexamic acid has been investigated as a systemic treatment option, particularly in patients with persistent disease [2], and tranexamic acid has also been administered through different routes, including oral, topical, and intradermal approaches [3]. Comparative evidence from randomized controlled trials demonstrates that numerous therapeutic modalities have been evaluated, with variable treatment responses across interventions [4]. Refractory melasma refers to persistent melasma that demonstrates an inadequate response to appropriately selected conventional therapies and remains particularly challenging to manage [5]. Such cases may require individualized treatment strategies based on previous therapeutic exposure, clinical characteristics, and contributing factors.

The clinical relevance of this case is considered within the context of orofacial harmonization. In addition to her dental qualifications, the treating author has completed additional professional training and certification in Oro Facial Harmonization, which incorporates evaluation of the perioral and facial tissues as part of a comprehensive approach to facial esthetics. Although melasma is primarily a dermatologic condition, facial skin appearance may represent an important component of the overall esthetic assessment in patients seeking comprehensive facial harmonization.

NewMelan (Krulig International Inc., Pembroke Pines, Florida, United States) is a multicomponent topical depigmenting system that has recently been described in a published case involving refractory centrofacial melasma [6]. The formulation includes depigmenting and antioxidant agents such as arbutin, kojic acid, azelaic acid, niacinamide, and phytic acid. Arbutin and kojic acid are proposed to reduce melanogenesis through inhibition of tyrosinase, whereas azelaic acid may suppress abnormal melanocyte activity and melanin synthesis. Niacinamide may decrease melanosome transfer from melanocytes to keratinocytes, while phytic acid provides antioxidant and metal-chelating effects that may further reduce tyrosinase-dependent pigmentation. NewMelan PRO (Krulig International Inc., Pembroke Pines, Florida, United States) is intended to produce controlled superficial exfoliation and facilitate the removal of melanin-containing keratinocytes, whereas NewMelan Day & Night (Krulig International Inc., Pembroke Pines, Florida, United States) provides continued topical depigmenting therapy during the active and maintenance phases. These proposed mechanisms are based on the known activities of the individual ingredients; however, evidence concerning the combined NewMelan formulation remains limited and does not establish superiority over established melasma treatments. Photoprotection remains an essential component of melasma management because ultraviolet and visible light exposure can contribute to pigmentation and recurrence [7]. This case report describes a patient with persistent facial melasma and substantial occupational sun exposure who reported unsatisfactory outcomes following multiple previous therapies and subsequently demonstrated short-term clinical improvement during a structured topical NewMelan protocol. The case is presented to document the clinical course of an individual patient rather than to establish comparative efficacy or superiority of the product.

## Case presentation

A 37-year-old medically healthy woman with no known drug allergies presented for evaluation of persistent facial hyperpigmentation. Clinical examination demonstrated a centrofacial pattern characterized by symmetric brown hyperpigmented macules and patches involving the forehead, bilateral malar regions, nasal bridge, and upper lip, with irregular borders and no alteration of the skin surface. The patient had Fitzpatrick skin phototype IV. A Wood's lamp examination was performed; however, the specific findings were not recorded. Dermoscopic examination was not performed. Based on the clinical morphology and distribution, the pigmentation was considered clinically consistent with melasma.

Relevant differential diagnoses included post-inflammatory hyperpigmentation, solar lentigines, drug-induced hyperpigmentation, lichen planus pigmentosus, pigmented contact dermatitis (Riehl's melanosis), exogenous ochronosis, and other melanocytic or pigmented lesions. These were considered less consistent with the overall presentation based on the symmetric centrofacial distribution, morphology, absence of preceding inflammatory skin changes or surface alteration, clinical history, and overall clinical examination. The patient worked as a hostess in a beach environment and reported frequent occupational sun exposure. The persistent facial pigmentation was a significant esthetic concern and, according to the patient, had negatively affected her self-confidence, particularly because her occupation involved frequent interaction with the public.

The patient reported a history of multiple previous treatment attempts for the facial pigmentation. These included chemical peels and laser-based procedures, which had not produced satisfactory clinical improvement. She subsequently underwent topical depigmenting therapy containing hydroquinone, tretinoin, and niacinamide, also without satisfactory resolution. The specific type and number of previous laser and chemical-peel sessions and the concentrations and duration of the topical therapies were not available for verification. According to the patient, some previously used topical products were associated with worsening of the pigmentation. She additionally reported previous use of systemic medication in an attempt to manage the condition; however, the specific medication, dosage, and duration were unavailable for verification. Persistent pigmentation despite these previous interventions prompted further evaluation.

A detailed medical history was obtained before initiation of treatment. The patient reported no known hormonal disorder or other relevant systemic condition. Based on the clinical presentation, previous treatment history, and persistent nature of the pigmentation, an individualized topical regimen using NewMelan PRO and NewMelan Day & Night was selected. No concurrent chemical peel, laser-based treatment, microneedling, injectable procedure, or additional depigmenting agent was incorporated into the treatment protocol. Given the patient's substantial occupational sun exposure, continued photoprotection was emphasized as an important component of management.

Treatment was initiated with NewMelan PRO, which was applied as a thin, uniform layer over the entire face without vigorous rubbing and remained in place for four hours. The application was repeated the following day. On the first night of treatment, NewMelan Day & Night was applied as a thin layer over the entire face and left in place overnight. Beginning on the third day, NewMelan Day & Night was applied three times daily for 15 days. The application frequency was subsequently reduced to twice daily for an additional 15 days, followed by once daily nighttime application as maintenance therapy. During the active treatment period, the patient was instructed not to combine the regimen with other topical products, moisturizers, or makeup. At baseline, persistent facial hyperpigmentation was clinically evident over the malar and adjacent facial regions despite the patient's history of multiple previous treatment modalities (Figure 1).

**Figure 1:**
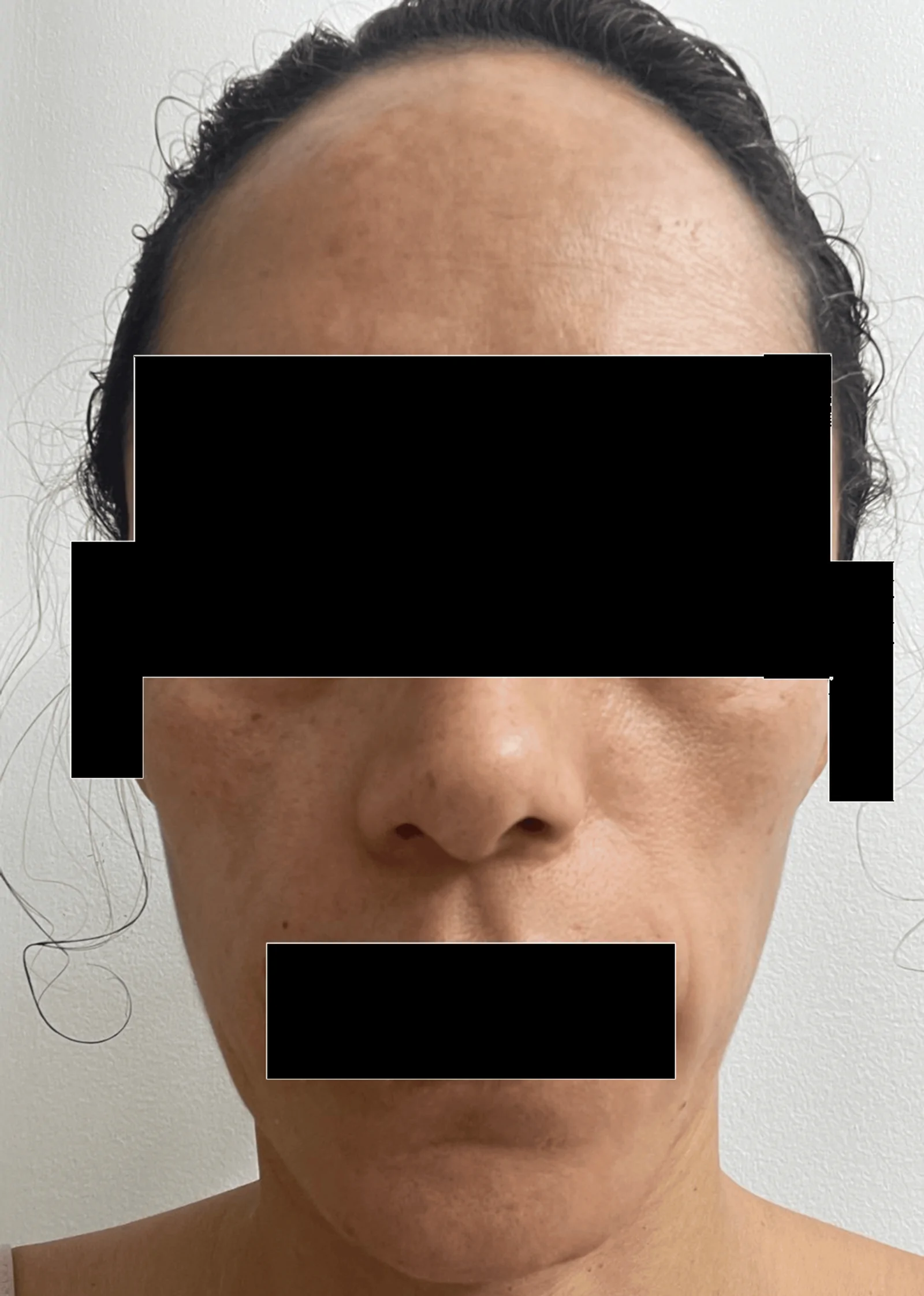
Initial Clinical Presentation Following Multiple Unsuccessful Melasma Treatments. Initial clinical presentation demonstrating persistent facial hyperpigmentation clinically consistent with melasma following multiple previously unsuccessful treatment modalities, including chemical peels, laser-based procedures, and topical depigmenting therapy. This photograph was obtained before initiation of the NewMelan topical protocol.

After initiation of the NewMelan protocol, progressive visible improvement was observed. At approximately 10 days, while active treatment was still ongoing, the facial pigmentation appeared visibly reduced in intensity and extent compared with the initial presentation (Figure 2). At this stage, the patient had not yet completed the subsequent phases of the treatment protocol.

**Figure 2:**
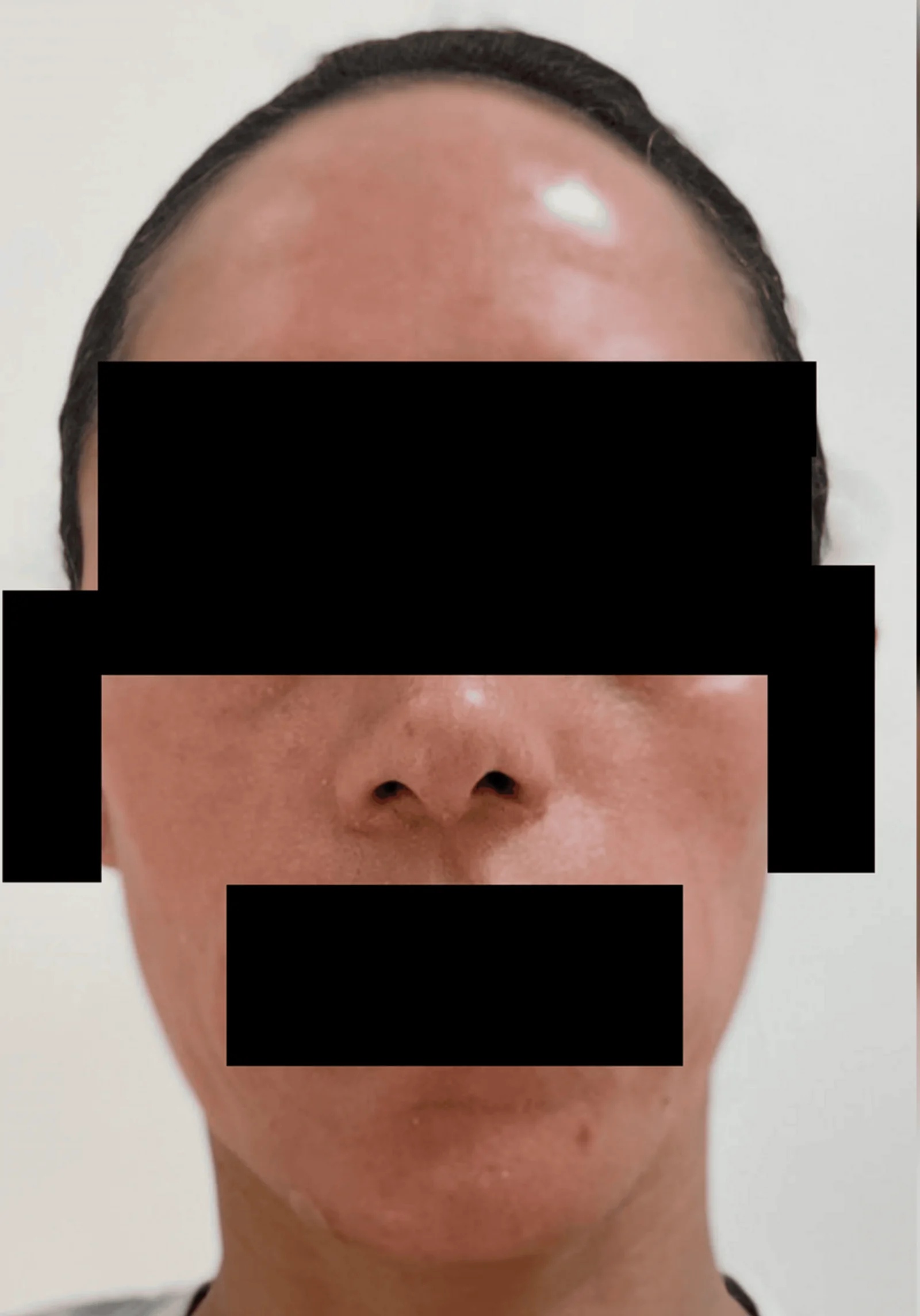
Clinical Appearance at Approximately 10 Days During the NewMelan Treatment Protocol. Clinical appearance approximately 10 days after initiation of the NewMelan topical protocol, demonstrating visible improvement in facial hyperpigmentation compared with the initial presentation. Treatment was ongoing at the time this photograph was obtained, and the patient had not yet completed the full treatment and maintenance protocol.

At the final documented clinical assessment, 15 days after treatment initiation, further visible improvement was observed, with decreased apparent pigmentation and a more homogeneous appearance of the affected facial regions (Figure 3). No recurrence or rebound pigmentation was observed during this documented follow-up period.

**Figure 3:**
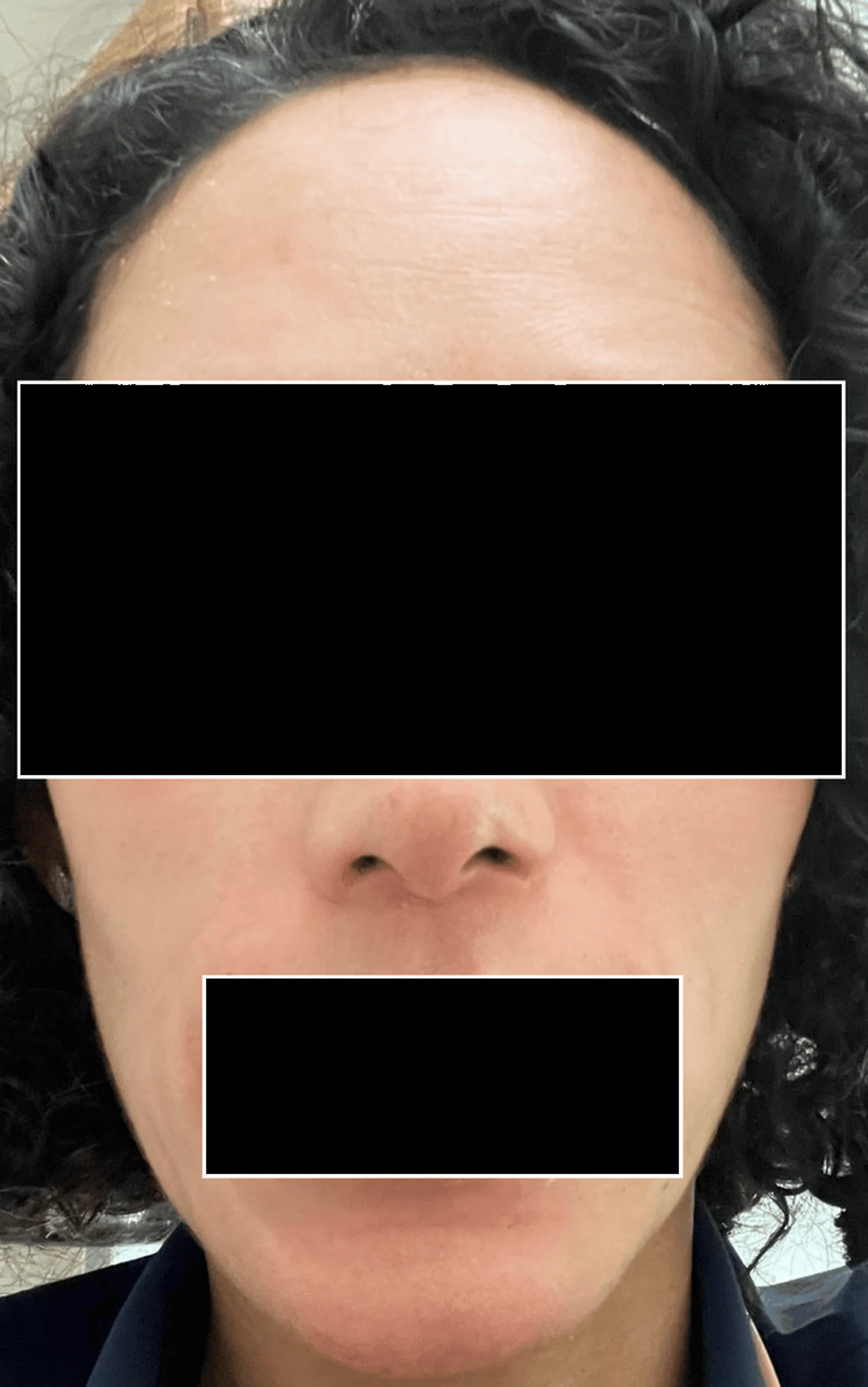
Clinical Appearance at the Final 15-Day Follow-Up Assessment. Clinical appearance at the final documented 15-day assessment, demonstrating further visible improvement in facial hyperpigmentation and a more homogeneous facial appearance. The patient continued topical NewMelan therapy thereafter as part of the treatment and maintenance regimen, together with continued photoprotection.

The patient subsequently transitioned to once daily nighttime application of NewMelan Day & Night as maintenance therapy together with continued photoprotection. She reported high satisfaction with the observed clinical improvement. Clinical photographs were obtained to document the treatment course. However, the photographs were acquired under different lighting, positioning, camera distance, and environmental conditions. Consequently, direct quantitative comparison of pigmentation based solely on the photographs was not performed. In addition, validated objective measures of melasma severity or pigmentation, such as the Melasma Area and Severity Index (MASI), colorimetry, or spectrophotometry, were not available for this case. The observed outcome was therefore characterized as clinical improvement rather than complete resolution or cure. This individual case report was not submitted to a local Institutional Review Board (IRB) or ethics committee; therefore, no IRB approval code was available. Written informed consent was obtained from the patient for publication of the clinical information and accompanying facial photographs.

## Discussion

The pathogenesis of melasma is complex and extends beyond increased melanogenesis alone. Current evidence suggests involvement of multiple interacting biological mechanisms and alterations within the cutaneous microenvironment, which may contribute to persistence and recurrence [8]. More recent analyses similarly emphasize the multifactorial nature of melasma and the interaction of environmental, hormonal, cellular, and molecular factors in its development [9].

The present case is notable because the patient reported persistent facial melasma despite previous treatment with chemical peels, laser-based procedures, and topical agents containing hydroquinone, tretinoin, and niacinamide. Chemical peels remain an established therapeutic option for selected patients with melasma, although treatment response and tolerability vary and careful patient selection is necessary [10]. Evidence-based reviews also support the use of topical depigmenting therapies while emphasizing that no single therapeutic approach is universally effective for all patients [11]. More recent reviews have expanded the therapeutic landscape to include established and emerging topical, systemic, and procedural approaches [12].

Topical and systemic therapies therefore continue to play important roles in melasma management, with treatment selection influenced by disease severity, previous therapeutic exposure, tolerability, and individual patient characteristics [13]. A multimodal and stepwise treatment strategy has been advocated because the complex pathogenesis and recurrent nature of melasma may limit the effectiveness of isolated interventions [14].

Laser-based procedures represent another therapeutic option, particularly in selected patients with persistent pigmentation, but their use requires careful consideration because response may vary and pigmentary complications can occur [15]. Broader management recommendations similarly emphasize individualized therapy, maintenance strategies, and photoprotection because recurrence following initial improvement remains a major clinical concern [16]. Fractional laser treatment combined with topical therapy has also been investigated in melasma resistant to triple combination cream, illustrating the potential role of procedural approaches in selected refractory cases [17].

In the present patient, progressive visible improvement was observed following initiation of the structured NewMelan topical protocol, with noticeable reduction in pigmentation at approximately 10 days and further improvement during continued treatment. Importantly, no concurrent laser therapy, chemical peeling, microneedling, injectable procedure, or additional depigmenting agent was used during this period. Comprehensive reviews of melasma emphasize the heterogeneity of therapeutic response and the importance of individualized treatment selection [18]. Therefore, the temporal association observed in this patient supports only that improvement occurred during use of the topical protocol; it does not establish causality, comparative efficacy, or superiority over established treatments.

Microneedling has also been investigated as an adjunct to topical therapies and may enhance treatment response in selected patients, although available evidence does not establish it as universally appropriate for all cases [19]. Natural products represent another area of investigation, with recent systematic review evidence evaluating their potential efficacy in melasma treatment [20]. Together, the breadth of available therapeutic approaches illustrates both the continuing interest in melasma treatment and the difficulty of identifying a universally effective strategy.

Photoprotection was particularly relevant in the present case because the patient reported substantial occupational sunlight exposure associated with working in a beach environment. Continued photoprotection and reduction of avoidable sun exposure were therefore emphasized throughout treatment and maintenance. Maintenance therapy was similarly considered important because visible improvement does not necessarily represent permanent resolution and recurrent pigmentation remains a concern in melasma.

The patient's reported improvement in self-confidence and high satisfaction with the clinical outcome are also clinically relevant. Because melasma affects highly visible facial areas, patient perceived improvement may be an important component of clinical outcome assessment. Nevertheless, subjective satisfaction cannot substitute for validated objective assessment of pigmentation severity.

From an orofacial esthetic perspective, this case illustrates the importance of considering the facial soft tissues and skin appearance together with oral and dental esthetics when evaluating overall facial harmony. However, melasma remains primarily a dermatologic condition, and this report should not be interpreted as suggesting that the management of melasma constitutes routine general dental practice. The present case was managed in the context of the author's additional training in Oro Facial Harmonization. Dermatologic or medical referral remains appropriate when the diagnosis is uncertain, the presentation is atypical, or management extends beyond the practitioner's professional training and scope.

Several limitations should be considered when interpreting this case. First, this report describes a single patient and therefore cannot be generalized to the broader population of patients with melasma. Second, clinical photographs were obtained under non-standardized conditions with differences in lighting, positioning, distance, and environmental exposure, limiting direct photographic comparison. Third, validated objective measurements such as the Melasma Area and Severity Index (MASI), colorimetry, or spectrophotometry were not available. Fourth, the observation period documenting the initial response was short, and longer follow-up is required to determine whether the observed improvement is maintained or whether recurrence occurs. Finally, details regarding some previous systemic treatment were unavailable for verification, limiting complete characterization of the patient's prior therapeutic history. Detailed tolerability and adverse-event data were not systematically recorded during the treatment course; therefore, the safety and tolerability of the protocol cannot be adequately assessed from this single case.

Despite these limitations, this case documents short-term clinical improvement of persistent facial melasma during a structured topical NewMelan regimen following multiple previously unsuccessful treatment modalities. The findings should be interpreted as an individual clinical observation rather than evidence of comparative effectiveness. Further prospective studies using standardized photography, validated pigmentation severity measures, longer follow-up, and appropriate comparison groups would be necessary to evaluate the effectiveness, tolerability, durability, and potential role of this topical protocol in the broader management of melasma.

## Conclusions

This case describes short-term clinical improvement in persistent facial melasma following a structured NewMelan topical protocol in a patient who reported unsatisfactory outcomes with multiple previous treatment modalities. Progressive visible improvement was observed during treatment, accompanied by high patient satisfaction. However, these findings represent a single-patient clinical observation and should not be interpreted as evidence of comparative efficacy or superiority over established melasma therapies.

The case highlights the importance of individualized treatment planning, careful evaluation of previous therapies and contributing environmental factors, continued photoprotection, and maintenance therapy in the management of persistent melasma. Longer follow-up and prospective studies using standardized photography and validated pigmentation assessment methods are needed to determine the effectiveness, safety, and durability of this topical protocol.

## References

[1] Neagu N, Conforti C, Agozzino M (2022). Melasma treatment: a systematic review. J Dermatolog Treat.

[2] Bala HR, Lee S, Wong C, Pandya AG, Rodrigues M (2018). Oral tranexamic acid for the treatment of melasma: a review. Dermatol Surg.

[3] Konisky H, Balazic E, Jaller JA, Khanna U, Kobets K (2023). Tranexamic acid in melasma: a focused review on drug administration routes. J Cosmet Dermatol.

[4] Liu Y, Wu S, Wu H, Liang X, Guo D, Zhuo F (2021). Comparison of the efficacy of melasma treatments: a network meta-analysis of randomized controlled trials. Front Med.

[5] Yao H, Shen S, Gao X, Feng J, Song X, Xiang W (2024). Definition of refractory melasma and its treatment: a review. Lasers Med Sci.

[6] Sanchez Meza MC (2026). Treatment of refractory centrofacial melasma in a 64-year-old female using the NewMelan Depigmentation System: a case report. Cureus.

[7] Morgado-Carrasco D, Piquero-Casals J, Granger C, Trullàs C, Passeron T (2022). Melasma: the need for tailored photoprotection to improve clinical outcomes. Photodermatol Photoimmunol Photomed.

[8] Artzi O, Horovitz T, Bar-Ilan E (2021). The pathogenesis of melasma and implications for treatment. J Cosmet Dermatol.

[9] Ali L, Al Niaimi F (2025). Pathogenesis of melasma explained. Int J Dermatol.

[10] Sarkar R, Lakhani R (2024). Chemical peels for melasma: a systematic review. Dermatol Surg.

[11] McKesey J, Tovar-Garza A, Pandya AG (2020). Melasma treatment: an evidence-based review. Am J Clin Dermatol.

[12] Gan C, Rodrigues M (2024). An update on new and existing treatments for the management of melasma. Am J Clin Dermatol.

[13] Sarkar R, Handog EB, Das A (2023). Topical and systemic therapies in melasma: a systematic review. Indian Dermatol Online J.

[14] Philipp-Dormston WG (2024). Melasma: a step-by-step approach towards a multimodal combination therapy. Clin Cosmet Investig Dermatol.

[15] Campos V, Kalil C, Zagonel L (2026). Lasers for melasma. Dermatol Clin.

[16] Suggs AK, Hamill SS, Friedman PM (2018). Melasma: update on management. Semin Cutan Med Surg.

[17] Tourlaki A, Galimberti MG, Pellacani G, Bencini PL (2014). Combination of fractional erbium-glass laser and topical therapy in melasma resistant to triple-combination cream. J Dermatolog Treat.

[18] Ogbechie-Godec OA, Elbuluk N (2017). Melasma: an up-to-date comprehensive review. Dermatol Ther (Heidelb).

[19] Bailey AJ, Li HO, Tan MG, Cheng W, Dover JS (2022). Microneedling as an adjuvant to topical therapies for melasma: a systematic review and meta-analysis. J Am Acad Dermatol.

[20] Morais MT, Gonçalves TL, de Paula Soares L, Sato AF, Oliveira Júnior RG, Silva Almeida JR (2026). Efficacy of natural products in the treatment of melasma: a systematic review. Biomed Pharmacother.

